# Editor’s introduction to this issue (G&I 17:4, 2019)

**DOI:** 10.5808/GI.2019.17.4.e35

**Published:** 2019-12-23

**Authors:** Taesung Park

**Affiliations:** Department of Statistics, Seoul National University, Seoul 08826, Korea

In this issue, there are 13 articles: four Review Articles, five Original Articles, and one article each in the categories of Application Notes, Clinical Genomics, Genome Archive, and Opinion. The first review, by Seo et al. (Korea National University of Education, Korea), deals with the contribution of long non-coding RNA to cancer development. The authors provide a thorough review of the oncogenic role of linc00152 in tumorigenesis, as well as an overview of recent clinical studies on the effects of linc00152 expression in human cancers.

The second review article, by M. Kim (Korea Research Institute of Bioscience & Biotechnology, Korea), presents a thorough review of the role of DNA methylation as a potential biomarker of type 2 diabetes (T2D), via a summary of case-control studies on the DNA methylome of T2D and a discussion of the possibility of DNA methylation as both a cause and consequence of T2D.

The third review, by S. Lee (Sejong University, Korea), is about statistical methods for survival analysis using genomic data. She provides an excellent review of more advanced survival analysis models for high-dimensional omics data, including regularization. She also reviews modern machine learning approaches to survival analysis, which fit nonlinear and complex interaction effects between predictors, resulting in more accurate predictions of the probability of individual survival.

The fourth review is by Y. Chung (Kyonggi University, Korea), and deals with recent advances in Bayesian inference of isolation-with-migration (IM) models for explaining population divergence in the presence of migrations. She presents an informative review of the Bayesian methods commonly used to estimate IM models and compares differences among these inference methods.

This issue also contains five original articles. First, Lee et al. (Hanyang University, Korea) present an analysis of unmapped regions (UMRs) associated with long deletions in Korean whole genome sequences based on short read data. The authors developed a program to select UMR long deletion candidates from short read sequencing data. Testing 40 Korean genomes, the authors could detect about 80% of UMR long deletions by comparing the candidates with the long deletion breakpoints contained in the genomes available from the 1000 Genomes project. Their approach could be useful for practical purposes in studies on long deletions utilizing short read data.

Lee and Hong (Seoul National University College of Medicine, Seoul) performed a functional annotation of germline de novo variants (DNVs) from healthy individuals. Using a large number of DNVs identified from the whole-genome sequencing of 1,902 healthy trios from the SFARI study and 20 healthy Korean trios, the authors showed that nonpathogenic DNVs were enriched in functional elements of the genome, but relatively depleted in regions of common copy number variants.

Park et al. (Seoul National University, Korea) present a method to determine the pure additive contribution of genetic variants such as single-nucleotide polymorphisms (SNPs) in prediction models for various diseases. Since most prediction models include both demographic variables and SNPs, it is difficult to evaluate the pure additive contribution of genetic variants. Using propensity score matching, the authors successfully evaluated the pure additive contribution of SNPs to T2D in the Korean population.

Choi et al. (Center for Genome Science, Korea) performed validation studies for 422 variants associated with glycemic indices, liver enzyme levels, and T2D in 125,872 samples from the Korean population. Among 422 independently associated variants, 284, 320, and 361 variants were replicated in the Korean population, the European population, and either of the two populations, respectively. However, 61 variants were replicated in neither Koreans nor Europeans. The effect sizes in Koreans and Europeans were shown to be modestly correlated. The authors emphasized that these differences in effect sizes and statistical significance among ancestry groups should be well accounted for when constructing polygenic risk scores for prediction.

The final research article is by M. Daoud, who presents an extension of the largest generalized-eigenvalue based distance metric in arbitrary feature spaces to classify composite data points, such as heterogeneous sets of biosequences. The author analyzed the impact of linear and non-linear transformation functions on classifying and clustering collections of heterogeneous sets of biosequences.

In this issue, there is one application note. Mok and Park (Seoul National University, Korea) present the hierarchical structural component model for pathway analysis of gene expression data (HisCoM-PAGE) software for performing pathway analysis of gene expression data using hierarchical structural component models. The HisCoM-PAGE software can be applied to various types of gene expression data, such as microarray or RNA-seq data.

The article on clinical genomics by Min et al. (The Catholic University of Korea, Korea) presents a validation study showing that the *KRAS* mutations identified from colorectal cancer tumor tissues were consistently detected in plasma cell-free DNA samples from three colorectal cancer patients by digital polymerase chain reaction.

The article in the Genome Archives section by Sulthana et al. (Genome Valley, Telangana, India) provides a high-quality draft genome and characterization of the commercially potent probiotic lactobacillus strains *Lactobacillus acidophilus* UBLA-34, L. *paracasei* UBLPC-35, *L. plantarum* UBLP-40, and *L. reuteri* UBLRU-87.

Finally, the opinion article by H. Kim (Korea Research Institute of Bioscience and Biotechnology, Korea) discusses the significance of artificial intelligence technology and big data in the biosciences and ubiquitous robotic companions in the fourth industrial revolution. The author concludes that the introduction of automated robots in this field is not just for our convenience, but is a prerequisite for artificial intelligence in the real sense and the consequent accumulation of basic scientific knowledge.

